# Adventitious Root Formation in Cuttings: Insights from *Arabidopsis* and Prospects for Woody Plants

**DOI:** 10.3390/biom15081089

**Published:** 2025-07-28

**Authors:** Peipei Liu, Shili Zhang, Xinying Wang, Yuxuan Du, Qizhouhong He, Yingying Zhang, Lisha Shen, Hongfei Hu, Guifang Zhang, Xiaojuan Li

**Affiliations:** 1 State Key Laboratory of Efficient Production of Forest Resources, Beijing Forestry University, Beijing 100083, China; peipeiliu@bjfu.edu.cn (P.L.); zhangshili@bjfu.edu.cn (S.Z.); wangxinying@bjfu.edu.cn (X.W.); heqizouhong@bjfu.edu.cn (Q.H.); zhangyingying@bjfu.edu.cn (Y.Z.); shenlisha@bjfu.edu.cn (L.S.); 2 Key Laboratory of Genetics and Breeding in Forest Trees and Ornamental Plants, Ministry of Education, College of Biological Sciences and Technology, Beijing Forestry University, Beijing 100083, China; 3 Nanchang Innovation Institute, Peking University, Nanchang 330096, China; hongfei.hu@pkuncii.cn

**Keywords:** adventitious root formation, age, auxin, cuttings, jasmonic acid, vegetative reproduction

## Abstract

Cutting propagation is a commonly employed technology for vegetative reproduction in agricultural, forestry, and horticultural practice. The success of cutting propagation depends on adventitious root (AR) formation—a process whereby roots regenerate from stem cuttings or leaf cuttings. In this review, we summarize the distinct stages of cutting-induced AR formation and highlight the pivotal roles of plant hormones and age in this process. Jasmonic acid (JA) acts as a master trigger for promoting AR formation, while auxin serves as the core regulator, driving AR formation. Furthermore, plant age is a crucial factor determining the regenerative competence of cuttings. Notably, age and JA collaboratively modulate auxin synthesis in cutting-induced AR formation. Overall, this review not only elucidates the molecular mechanisms underlying AR formation but also provides valuable insights for improving efficiency of cutting propagation in various plant species.

## 1. Introduction

Asexual reproduction is a core technology for rapidly propagating elite germplasm in agriculture, forestry, and horticulture [1]. This technique preserves the desirable traits of parent trees, significantly shortens the breeding cycle, and exhibits a high genetic stability [2]. Cuttings, a vital asexual propagation technique, play a vital role in large-scale propagation. Adventitious rooting (AR) is a key step for cutting propagation, and this process is a form of de novo root regeneration (DNRR) [3].

The capacity for AR formation shows significant variation across different species and even among varieties of the same species. For example, *Pinus tabuliformis*, *Paeonia suffruticosa*, and *Camellia oleifera* continue to pose challenges in propagation through cuttings [4,5]. In production practice, ARs from cuttings are often classified into three types: cortical rooting, callus rooting, and mixed rooting [6]. Cortical rooting, also known as the “easy-to-root” type, arises directly from the explants and primarily originates from the vascular cambium and surrounding pith rays, such as *Petunia hybrid* [7,8]. Callus rooting, categorized as the “difficult-to-root” type, is an indirect process that requires the initial formation of callus prior to the development of AR primordia, such as *Pinus* spp. [9]. Mixed rooting combines both cortical and callus rooting types, which predominates under natural conditions such as Chinese pepper (*Zanthoxylum beecheyanum* K. Koch) [10].

Recent advances have gradually elucidated the regulatory mechanisms underlying AR formation. In this review, we summarize the process of cutting-induced AR formation, the regulatory mechanisms involved in this process by phytohormones (especially jasmonate (JA) and auxin), and the potential age-related regulatory pathways. This provides a theoretical foundation for understanding the complex regulatory mechanisms of cuttings and establishes a molecular basis for developing highly efficient cutting techniques.

## 2. Cutting-Induced AR Formation

The framework of DNRR can be divided into three phases, which are early signaling, auxin accumulation, and cell fate transition [3,11]. Among them, the first phase involves changes in auxin biosynthesis in the converter cells. In the second phase, auxin is transported to vascular stem cells with regenerative capacity. In the third phase, auxin triggers the cell fate transition in regeneration-competent cells to form ARs [12].

Early signaling triggers AR formation during cutting propagation. The early signals include wound signals, developmental signals, and environmental signals [3,13,14,15]. Wound signaling has short- and long-term effects on leaf explants. Short-term wound signals include changes in plasma membrane potential, increased intracellular Ca^2+^ concentration and H_2_O_2_ production, and the action of phytohormones such as JA, salicylic acid (SA), and ethylene [3]. In the study of biochemical and anatomical parameters during the cutting process of the recalcitrant *Eucalyptus globulus* Labill and the more easily rooted *Eucalyptus grandis* W.Hill ex Maiden, it was revealed that *E. grandis* exhibits higher cambial activity and more precise regulation of redox conditions. These characteristics may influence reactive oxygen species (ROS) signaling and phytohormone homeostasis in cuttings, thereby affecting AR formation [16]. Long-term wound signals may activate the expression of genes like *petunia NAM* and *Arabidopsis ATAF1*, *ATAF2*, and *CUC2* (*NAC1*) and *YUCCA4* (*YUC4*), which regulate the cellular environment and maintain auxin levels to facilitate AR emergence [17]. Environmental signals such as dark treatment and circadian rhythms may also enhance AR formation by affecting the expression of auxin synthesis genes [18]. Approximately 4 h after leaf explant detachment, auxin begins to be synthesized and accumulates in converter cells guided by early signals [19]. At approximately 12 h after wounding, auxin accumulates in regeneration-competent cells (e.g., procambium and vascular parenchyma cells) in the explant close to the wound site [20].

The cell fate transition phase is divided into four steps: priming, initiation, patterning, and emergence [3,11,21]. In the first step, auxin promotes the differentiation of regenerating-competent cells such as procambium and some vascular parenchyma cells into root founder cells about 1–2 days after leaf explant isolation. Approximately 2–4 days after leaf explant isolation, root founder cells form a dome-shaped root primordium with several layers of cells by cell division. Subsequently, the root primordium undergoes successive cell divisions to form the apical meristematic tissue. Finally, in the ‘emergence’ stage, the AR tip forms and ultimately emerges through the epidermis of the leaf explant [3].

## 3. Key Factors in Cutting-Induced AR Formation

Currently, the efficiency of rooting in cuttings is improved mainly by rejuvenating or adding plant growth regulators. Rejuvenation alters the physiological age of explants—a critical determinant of rooting success [22]. Plant rejuvenation can be achieved in practice by artificial methods such as repeated grafting, continuous in vitro micropropagation, or root cuttings in succession [23,24,25]. In forest tree species, the decline in AR formation potential among stem cuttings is correlated with tree age and maturity [26]. In vitro shoot culture-induced rejuvenation is used in apple rootstocks through DNA methylation reprogramming to retain their juvenile state, restoring their competence for AR formation [27]. Consequently, regulating the physiological age of cutting material may effectively enhance rooting rates. Another crucial strategy for promoting de novo root regeneration in cuttings involves the application of plant growth regulators, among which auxin is recognized as the primary phytohormone regulating AR formation [28]. Different types of auxins and concentrations affect AR formation [29]. In addition, recent studies have shown that cutting diameter also affects AR formation [30]. In *Syzygium maire*, 1–2 mm softwood cuttings achieved 63.3% rooting without auxin, while supplementation with 1.5 g L^−1^ IBA raised the success rate to 75% and increased root number [31].

### 3.1. JA: A Master Trigger for AR Formation

JA serves as a central wound signal in promoting AR formation. Studies on petunia (*Petunia hybrida*) and pea (*Pisum sativum*) demonstrate that JA accumulates rapidly at the stem base, thereby promoting AR initiation [7,32,33]. Similarly, in thin cell layers of tobacco and *Arabidopsis* responsible for AR development, low JA concentrations enhance root formation [34]. In *P. ussuriensis*, *PuHox52* positively regulates AR formation by activating the JA signaling pathway [35]. In *Camellia sinensis*, aluminum, an essential element for root growth and development in acidic soil, can promote AR formation by activating genes involved in JA biosynthesis as well as genes related to auxin transporter proteins during root formation [36].

The mechanisms by which JA regulates AR formation have been extensively studied. JA activates the expression of *ERF115* and regulates the formation of ARs by promoting cytokinin biosynthesis [37]. In addition, JA promotes the expression of the *AUXIN-OXIDIZING DIOXYGENASE 1* (*DAO1*) gene, which regulates the feedback crosstalk between auxin and JA during AR initiation [38]. In *Arabidopsis* leaf explants, JA levels are highly induced, activating the transcription factor ETHYLENE RESPONSE FACTOR109 (ERF109). *ERF109* upregulates expression of an auxin biosynthesis-related gene, *ANTHRANILATE SYNTHASE α1* (*ASA1*), to stimulate AR induction [39]. *ERF109* promotes *ASA1* expression depending on an epigenetic modification mechanism involving histone H3 lysine 36 trimethylation (H3K36me3) at the *ASA1* locus prior to wounding [40]. This H3K36me3 mark is likely essential for the rapid JA-induced upregulation of multiple genes [40]. However, prolonged JA signaling can adversely affect root development. Therefore, two hours after wounding of leaf explants, the JA signaling pathway is blocked through the interaction between *ERF109* and jasmonate ZIM-domain (JAZ) proteins [40]. The wound-inducible transcription factor ENHANCER OF SHOOT REGENERATION1 (ESR1) in *Arabidopsis* leaf explants employs a dual mechanism to activate ASA1 expression, crucial for auxin-dependent de novo root organogenesis at wound sites. ESR1 initially interacts with *HISTONE DEACETYLASE6* (*HDA6*) to inhibit *JASMONATE-ZIM DOMAIN5* (*JAZ5*) expression via histone H3 deacetylation, enabling *ERF109* to activate *ASA1*. Additionally, *ESR1* directly binds to the promoter region of *ASA1* to enhance its expression, collectively maximizing local auxin biosynthesis and AR formation [41]. Overall, JA precisely modulates auxin dynamics via a complex regulatory network to coordinate efficient AR regeneration. Additionally, JA activates the melatonin (MT) receptors *SlPMTR1/2*, which are structurally analogous to auxin receptors. These receptors promote AR formation by transmitting signals through the *G-PROTEIN ɑ-SUBUNIT 1* (*SlGPA1*) to *SHOOTBORNE ROOTLESS 1* (*SlSBRL1*), a key regulator of wound-induced root regeneration [42].

During the root regeneration process in cuttings, detached leaves or stems encounter multiple stresses. Wounding and osmotic stress jointly induce *ABA INSENSITIVE5* (*ABI5*) expression and *MYC2* upregulation [43]. The two transcription factors ABI5 and MYC2 form a complex that directly binds to the *β-GLUCOSIDASE18* (*BGLU18*) promoter region, activating its expression. *BGLU18* functions to release active abscisic acid (ABA) from its glucose ester form, resulting in increased ABA accumulation, which in turn preserves AR formation under stress. Moreover, sequential application of JA and ABA enhances root regeneration in *Arabidopsis* and poplar cuttings [43]. While ROS, ethylene, and SA also act as early signaling molecules in this process [44], the wound signaling pathways by which they initiate AR formation require further investigation.

### 3.2. Age: The Key Determinant Controlling AR Formation

The rooting potential of cuttings is significantly affected by age, with a general decline in rooting efficiency as the plant matures. Rooting rates and AR quality were significantly higher in cherry (*Prunus avium*) juvenile cuttings than in mature cuttings [45]. MicroRNA 156 (miR156) serves as a critical determinant of plant age, with its high expression levels during the juvenile phase being positively correlated with enhanced AR formation ability [46,47,48]. In tomato (*Solanum lycopersicum*) and tobacco (*Nicotiana tabacum*), overexpression of the miR156 precursor gene promotes AR formation [47,49]. Studies in *Arabidopsis* reveal that miR156 expression diminishes as the plant matures, resulting in heightened expression of *SQUAMOSA PROMOTER BINDING PROTEIN-LIKE* (*SPL*) genes. Subsequently, *SPL2/10/11* repress the expression of AP2/ERF transcription factor, such as *ERF109*, curtailing wound-induced auxin production and undermining the plant’s AR formation capability [50].

*DEFICIENS-AGAMOUS-LIKE 1* (*DAL1*) serves as a pivotal regulator of age in gymnosperms, with its expression progressively increasing as age advances [51,52]. *DAL1* expression is lower in juveniles and increases with age. Research indicates that *LaDAL1* is highly expressed during the reproductive phase of *Larix species*. Heterologous overexpression of *LaDAL1* promotes seed germination, bolting, and flowering in *Arabidopsis thaliana* [53,54]. However, its specific role in regulating AR formation in plants remains to be elucidated. In studies on *Larix kaempferi*, LaAGL, an age-related transcription factor, shows markedly higher expression levels in the adult reproductive phase (25–50 years) compared to the juvenile vegetative phase (1–2 years). Pruning notably reduces the expression of *LaAGL2-2* and *LaAGL2-3* [55]. Nevertheless, the molecular mechanism by which *LaAGL* regulates AR formation is still unclear. Furthermore, small RNAs (sRNAs) differentially expressed during grafting in the *gymnosperm Sequoia sempervirens* may play a role in controlling rooting ability during plant rejuvenation [56]. These findings reveal that these genes act as age factors that may regulate plant AR formation.

### 3.3. Auxin: The Core Regulator Governing AR Formation

Both age and JA predominantly regulate AR formation through auxin-mediated mechanisms. Auxin, a core control factor, has been used to promote rooting since the late 1930s [57]. Indole-3-acetic acid (IAA), indole-3-butyric acid (IBA), and 1-naphthaleneacetic acid (NAA) are the primary active ingredients of rooting powders commonly used in plant cuttings [58,59]. In production practice, auxin application to cuttings is generally implemented via two distinct regimes: (i) short-duration, high-concentration quick-dips, and (ii) prolonged, low-concentration soaks. For example, 2000 mg L^−1^ IBA quick-dip maximally promotes rooting in *Paeonia* ‘Yang Fei Chu Yu’ cuttings (86.7% success) [60]. The 0.54 mM NAA treatment for 1 h effectively induced rooting in hybrid aspen (*Populus tremula* L. × *P.tremuloides* Michx.) single-node cuttings [29]. In white poplar (*Populus alba*) cuttings, IBA treatment induces root primordia and increases the number of ARs per cutting [61]. By applying synthetic auxin to the difficult-to-root species eucalyptus (*Eucalyptus × trabutii*) and apple (*Malus domestica*), their rooting of cuttings can be improved [62]. Together, these results illustrate the importance of auxin in enabling AR formation across diverse plant systems.

The molecular mechanisms by which auxin regulates AR formation have been extensively studied (Table 1). The initiation of AR formation depends on auxin signal perception and transduction. For example, *PagFBL1*, the poplar homolog of the auxin receptor TIR1, interacts with IAA28 to promote AR formation in the hybrid *P. alba × P. glandulosa* [63]. Conversely, the bZIP53 transcription factor negatively regulates AR formation by transcriptionally activating the expression of AUX/IAA genes *IAA4-1* and *IAA4-2*, which function as repressors of ARF activity in poplar [64]. In addition, studies combining quantitative trait loci (QTL) mapping and transcriptomic analysis of AR developmental traits in poplar hybrids have indicated that the genes *SUPERROOT2* (*SUR2*) and *TRYPTOPHAN SYNTHASE ɑ-CHAIN* (*TSA1*) may regulate AR formation by modulating IAA synthesis [65].

WUSCHEL-related homeobox (WOX) family members are crucial for auxin-mediated AR formation. In isolated *Arabidopsis* leaves, auxin activates *WOX11/12* to convert regenerative potential cells into root founder cells [12,19]. Subsequently, *WOX11/12* activates the transcription of *WOX5* and *WOX7*, enabling the transition of root founder cells into root primordia [66]. Acting synergistically, *WOX11/12* also directly activate *LBD16/29* transcription, which is essential for AR formation [67,68]. Similar regulatory functions of *WOX5* and *WOX11/12* have been identified in poplar. For example, overexpression of *PtoWOX11/12a* and *PtoWOX5a* markedly increases regenerated AR numbers [69]. Additionally, overexpression of *PtoWUSa*, another member of the WOX family, affects polar auxin transport, reducing root length while boosting root number [70]. In 84 K poplar, *PagWOX11* promotes DNRR by directly activating *PagLBD16* [71]. In apple (*Malus×domestica*), *MdTCP17* interacts with *MdWOX11*, inhibiting MdWOX11’s binding to the *MdLBD29* promoter and thus repressing AR primordium formation [72]. *WOX11* also regulates AR formation in rice (*Oryza sativa*) [73,74], *Panax ginseng* [75], and banyan (*Ficus macrocarpa*) [76]. These findings indicate that the AUXIN-WOX11/12-WOX5 and AUXIN-WOX11/12- LBD16/29 modules are vital for the cellular reprogramming underpinning AR formation across plant species.

MicroRNAs (miRNAs) are a class of short non-coding RNAs that can regulate AR formation by targeting key transcription factors such as ARFs [77]. The transcription factors ARF6, ARF8, and ARF17 have been identified as key regulators of AR formation, modulated by miR167 and miR160 in *Populus* [78,79]. MiR167 suppresses AR formation by targeting *ARF8* mRNA [78]. In contrast, miR160 promotes AR formation by regulating the activity of *ARF17* [79]. In addition, miR476a regulates AR formation by repressing *RESTORER OF FERTILITY* (*RFL*) genes (restricting mitochondrial energy production) [80]. It also activates PIN-FORMED2/5b (PIN2/5b) to promote auxin efflux, resulting in an increased number of ARs [81].

The function of auxin in AR formation has also been evidenced in other plants. In *Camellia sinensis*, the CsSPL9-CsGH3.4 module responds to auxin and negatively regulates AR formation by reducing free IAA concentrations [82]. In *Malus domestica*, *MdARF8*, an auxin-responsive factor, enhances AR formation by modulating the transcription of *GRETCHEN HAGEN 3* genes [15]. In contrast, BROAD-COMPLEX, TRAMTRACK AND BRIC A BRAC, and TRANSCRIPTION ADAPTOR PUTATIVE ZINC FINGER domain protein 2 (MdBT2) inhibit AR formation through interacting with *AUXIN RESPONSE FACTOR8* (*MdARF8*) and *INDOLE-3-ACETIC ACID INDUCIBLE3* (*MdIAA3*) [15]. In *Acer rubrum*, *ArAux/IAA13* and *ArAux/IAA16* were differentially expressed after IBA treatment, and they could interact with ARF proteins to regulate AR growth and development. Overexpression of *ArAux/IAA13* and *ArAux/IAA16* inhibited AR development, providing a molecular basis for improved rooting in *A. rubrum* [83]. Moreover, environmental factors such as light can modulate endogenous auxin homeostasis and signaling to govern AR formation. For example, blue light induces the most ARs in tea cuttings by elevating the levels of indole-3-carboxylic acid (ICA), ABA, JA, ABA-glucosyl ester, and trans-zeatin, and up-regulating key hormone-pathway genes (*YUC*, *AUX1*, *ARF*, *PIN1/3/4*, *PILS6/7*), revealing the molecular basis for rapid tea seedling propagation [84].

**Table 1:** List of auxin-associated genes involved in the formation of cutting-induced adventitious root.

Gene Name	Gene Family	Species	Roles in Adventitious Rooting	References
*PagFBL1*	*TIR1/AFB* receptor family	*Populus alba* × *P. glandulosa*	Auxin receptor, interacts with *IAA28* to promote AR formation	[63]
*IAA4-1*	*AUX/IAA* family	*Populus deltoides* and *P. euramericana*	Represses *ARF* activity; negatively regulates AR formation	[64]
*IAA4-2*	*AUX/IAA* family	*Populus deltoides* and *P. euramericana*	Represses *ARF* activity; negatively regulates AR formation	[64]
*AtWOX11*	*WUSCHEL*-related homeobox gene family	*Arabidopsis thaliana*	Auxin-induced; activates root founder cells	[68]
*AtWOX12*	*WUSCHEL*-related homeobox gene family	*Arabidopsis thaliana*	Auxin-induced; activates root founder cells	[68]
*AtWOX5*	*WUSCHEL*-related homeobox gene family	*Arabidopsis thaliana*	Activates the transition of root founder cells into root primordia	[66]
*AtWOX7*	*WUSCHEL*-related homeobox gene family	*Arabidopsis thaliana*	Activates the transition of root founder cells into root primordia	[66]
*AtLBD16*	*LBD* family	*Arabidopsis thaliana*	Directly activated by *AtWOX11/12*, essential for AR formation	[67]
*AtLBD29*	*LBD* family	*Arabidopsis thaliana*	Directly activated by *AtWOX11/12*, essential for AR formation	[67]
*PtoWUSa*	*WUSCHEL*-related homeobox gene family	*Populus tomentosa*	Alters polar auxin transport; reduces root length but increases AR number.	[70]
*PagWOX11*	*WUSCHEL*-related homeobox gene family	*Populus alba* × *P. glandulosa*	Activates *PagLBD16* to promote de novo root regeneration	[71]
*PagLBD16*	*LBD* family	*Populus alba* × *P. glandulosa*	Acts downstream of *PagWOX11* to mediate root regeneration	[71]
*MdTCP17*	*TCP* family	*Malus domestica* (apple)	Interacts with *MdWOX11* and blocks its binding to the *MdLBD29* promoter	[72]
*MdWOX11*	*WUSCHEL*-related homeobox gene family	*Malus domestica* (apple)	Activates *MdLBD29*; suppressed by *MdTCP17*	[72]
*AtARF6*	*ARF* family	*Arabidopsis*, *Populus*	Interacts with *WOX11* to activate *RGIs* and *LBD16* for adventitious root primordium	[68]
*AtARF8*	*ARF* family	*Arabidopsis*, *Populus*	Interacts with *WOX11* to activate *RGIs* and *LBD16* for adventitious root primordium	[68]
*miR167*	*microRNA family*	*Populus deltoides* × *Populus euramericana*	Suppresses AR formation by targeting ARF8 mRNA	[78]
*miR160*	*microRNA family*	*Populus deltoides* × *Populus euramericana*	Promotes AR formation by regulating the activity of ARF17	[79]
*miR476a*	microRNA family	*Populus tomentosa*	Represses *RFL* genes; activates *PIN2/5b* to promote auxin efflux and AR formation	[80]
*MdARF8*	*ARF* family	*Malus domestica* (apple)	Enhances AR formation by modulating *GH3* genes	[15]
*MdBT2*	*BTB-TAZ* family	*Malus domestica* (apple)	Inhibits AR formation by interacting with *MdARF8* and *MdIAA3*	[15]
*ArAuxIAA13*	*AUX/IAA* family	*Acer rubrum*	Repress AR formation through interaction with ARF proteins	[83]
*ArAuxIAA16*	*AUX/IAA* family	*Acer rubrum*	Repress AR formation through interaction with ARF proteins	[83]

## 4. Conclusions

**Figure 1:**
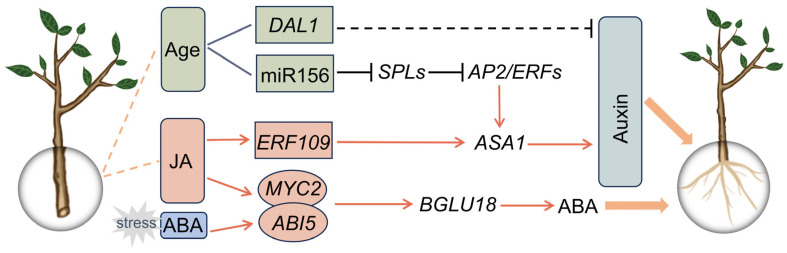
The molecular framework for hormone- and age-mediated adventitious root (AR) formation in cuttings. MicroRNA 156 (miR156), the hub regulator of juvenility, regulates the jasmonic acid (JA)-mediated wound signaling pathway, subsequently promoting auxin synthesis in angiosperms. *DAL1*, an age marker gene in gymnosperms, might be involved in AR formation by modulating auxin behavior. JA, acting as a wound signal, directly promotes auxin synthesis to enhance AR formation in cuttings. Under stress conditions, JA and ABA collaboratively amplify ABA signals to protect rooting in cuttings.

Cuttings are essential in asexual propagation of plants, characterized by their efficacy and reliability in conserving the parental plant’s favorable characteristics. Age and phytohormone are key factors influencing AR formation in cuttings. This review summarizes recent advances in the regulation of AR formation by auxin, JA, and age (Figure 1). We highlight how JA serves as a master trigger, while auxin acts as a core regulator, in cutting-induced AR formation in several species. Furthermore, we discuss the mechanistic basis of their interplay during AR formation. Most importantly, AR formation capacity decreases with increasing plant age and is regulated by miR156-SPL in *Arabidopsis*, whereas DAL1 in gymnosperms is a potential age factor that can be further investigated for its role in AR formation in the future. Interestingly, under stress conditions, JA and ABA collaboratively amplify ABA signals, ensuring the survival of cuttings in adverse environments. In practical production, for recalcitrant plants, cuttings can be pretreated with rejuvenation to reverse ontogenetic aging. Subsequently, exogenous JA or ABA application on rejuvenated cuttings can effectively enhance adventitious root formation. This offers a valuable strategy for large-scale plant propagation, enhancing the efficiency and feasibility of vegetative reproduction. Environmental factors also exert decisive control over adventitious root (AR) regeneration. In species such as *Pinus massoniana* [85], *Corylus avellana* L. [86], *Eucalyptus*, and *Populus* [87], clonal propagation is constrained by AR capacity, yet its efficiency can be markedly improved by precisely manipulating irradiance, temperature, mineral nutrition, and microbe–plant interactions. These exogenous signals converge with endogenous hormonal networks to determine AR competence. Future research is urgently needed to construct systems biology models covering environment-endogen synergistic regulatory mechanisms and integrate precision genome editing technologies such as CRISPR-Cas9 to modify key environmental response genes. In addition, synthetic biology tools, such as designing synthetic promoters or gene circuits, can be developed to finely regulate the expression of genes related to plant regeneration. These approaches are expected to break through the genotype-dependent bottleneck, thus establishing a high-throughput asexual reproduction technology system applicable to different species.

## Data Availability

No new data were created or analyzed in this study. Data sharing is not applicable to this article.
